# Supplementary material for: Co-Graft of Acellular Dermal Matrix and Split Thickness Skin Graft—A New Reconstructive Surgical Method in the Treatment of Hidradenitis Suppurativa
Source: Bioengineering (Basel). 2022 Aug 14;9(8):389. doi: 10.3390/bioengineering9080389 (PMC9404734; doi:10.3390/bioengineering9080389)
Supplement: Supplementary file 1 [file bioengineering-09-00389-s001.zip › bioengineering-1828357-supplementary final version after published.pdf]

bioengineering

Communication

Co-Graft of Acellular Dermal Matrix and Split Thickness Skin Graft - A New Reconstructive Surgical Method in the Treatment of Hidradenitis Suppurativa.

Marcin Gierek [1]*, Wojciech Łabuś [1], Anna Słaboń[1], Karolina Ziółkowska[1], Gabriela Ochała-Gierek[2], Diana Kitala[1], Karol Szyluk[3,4] and Paweł Niemiec [5]

Supplementary Material

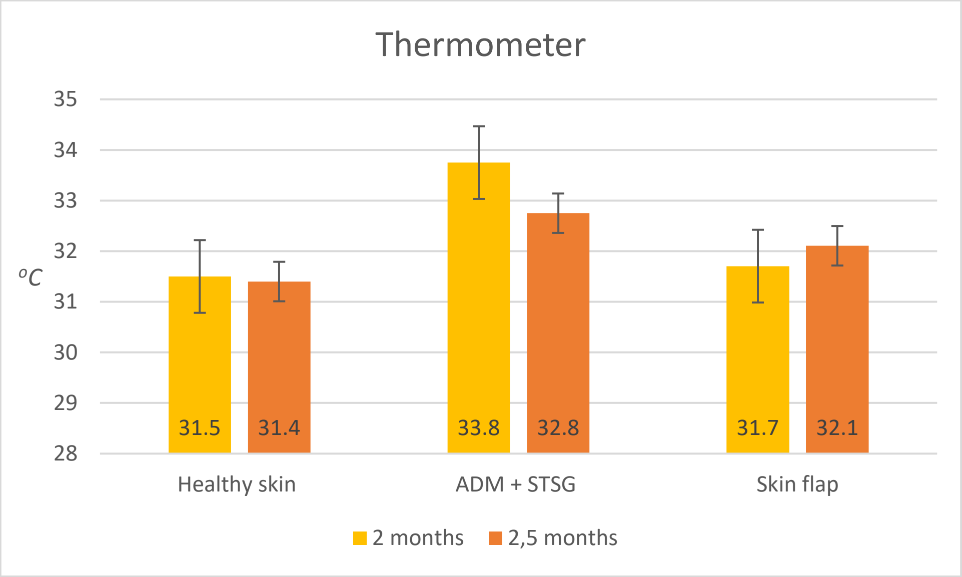

Figure S1. Temperature measurements of healthy skin, ADM + STSG and skin flap of case 1. Legend : ADM – Acellular Dermal Matrix, STSG – Split thickness skin graft.

The corneometer is made up of two electrodes with different electric charges, which create an electromagnetic field, on the basis of which the dielectric constant is calculated. The result from the corneometer is given in relative units, from 0 to 130.

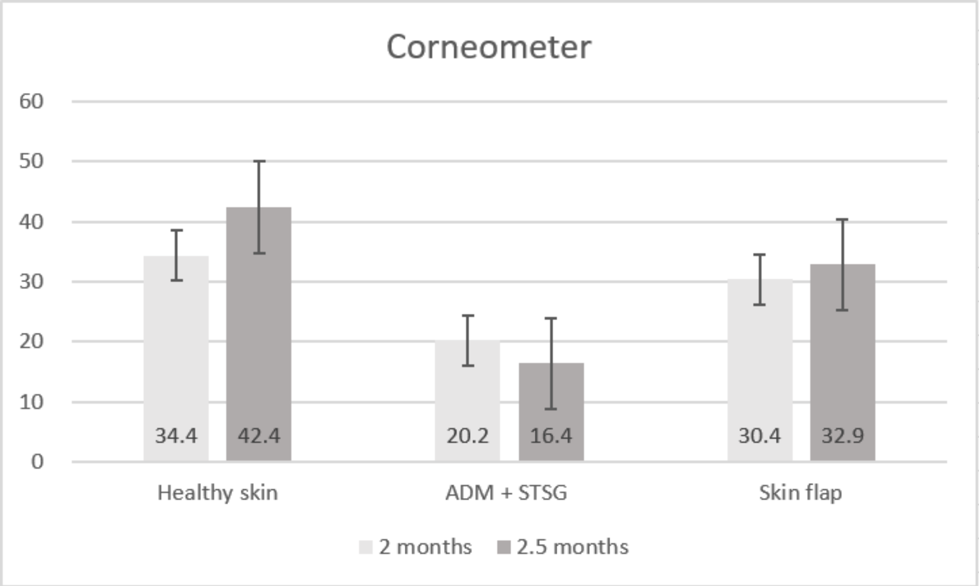

Figure S2. Corneometer measurements of healthy skin, ADM + STSG and skin flap of case 1. Legend : ADM – Acellular Dermal Matrix, STSG – Split thickness skin graft.

In the case of measuring redness, two different wavelengths are used to measure the skin's ability to absorb. One of these wavelengths refers to the spectral absorption peak of hemoglobin. The second wavelength was chosen to avoid the influence of a different color (e.g. bilirubin). The achieved results are displayed on the screen as indicators ranging from 0 to 999.

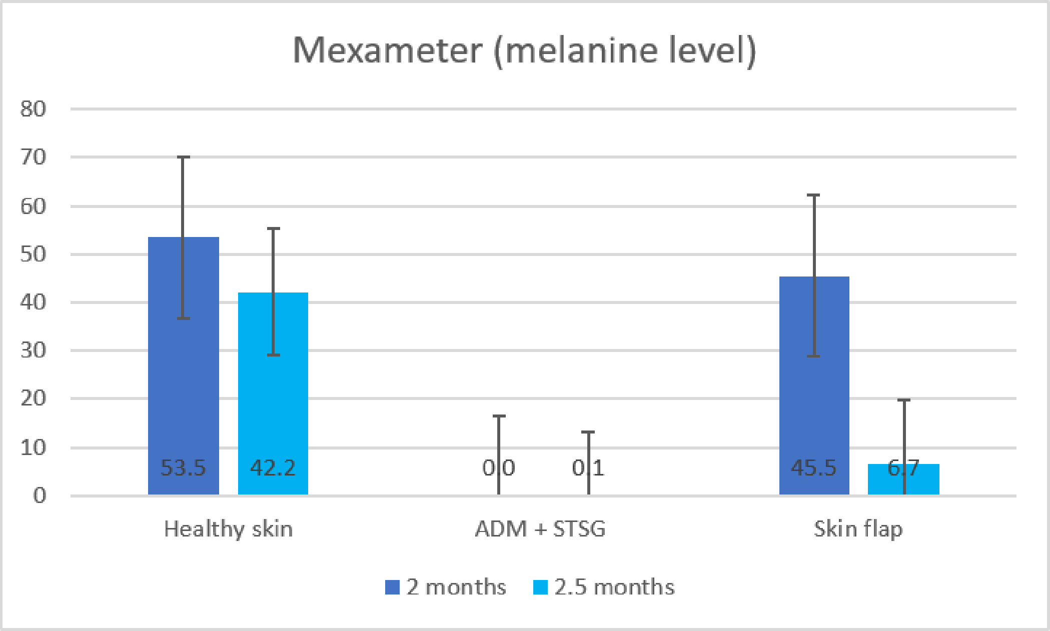

Figure S3. Mexameter (melanine level) measurements of healthy skin, ADM + STSG and skin flap of case 1. Legend : ADM – Acellular Dermal Matrix, STSG – Split thickness skin graft.

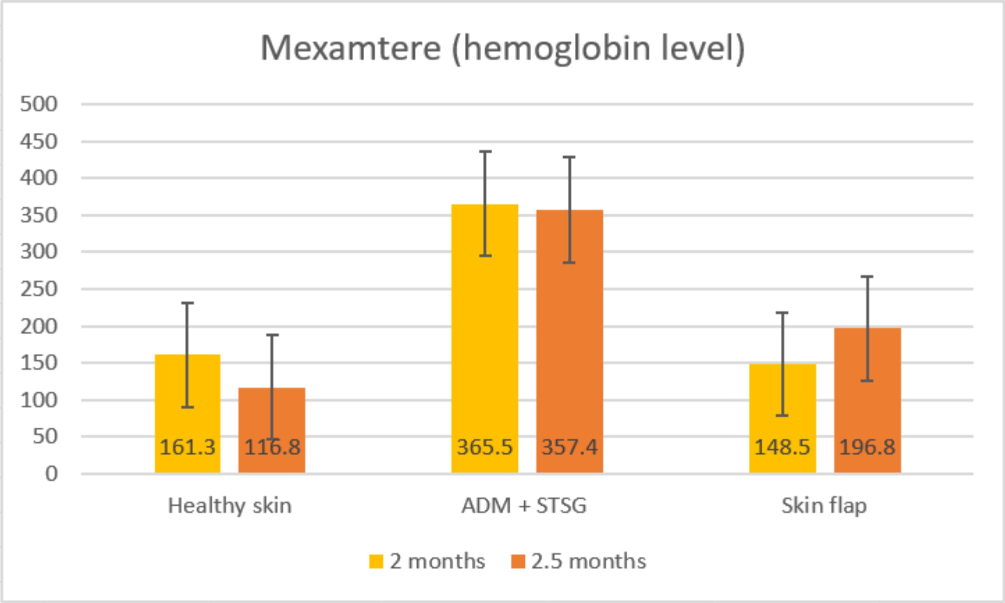

Figure S4. Mexameter (hemoglobine level) measurements of healthy skin, ADM + STSG and skin flap of case 1. Legend : ADM – Acellular Dermal Matrix, STSG – Split thickness skin graft.

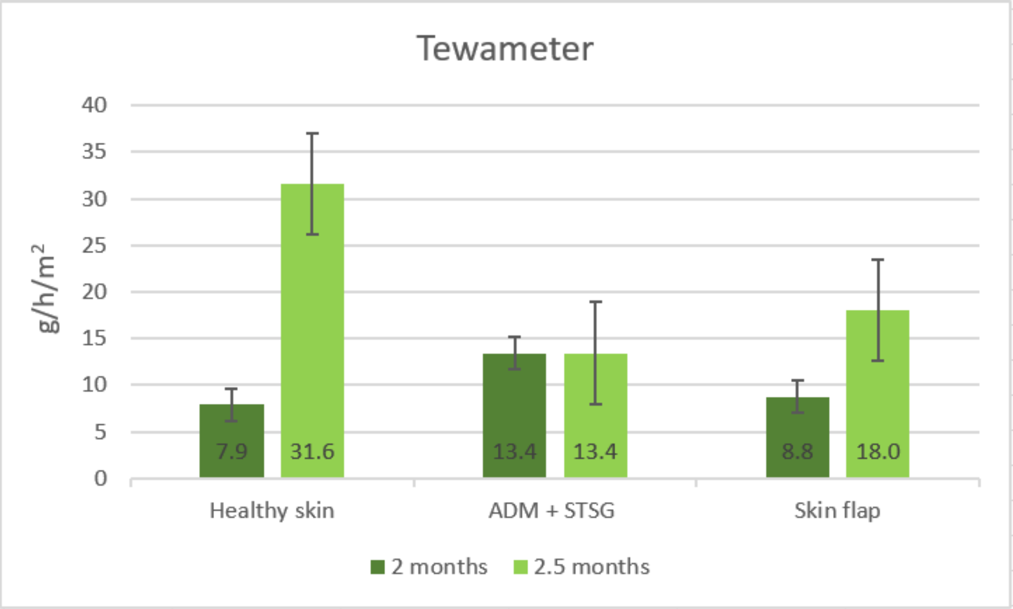

Figure S5. Tewameter measurements of healthy skin, ADM + STSG and skin flap of case 1. Legend : ADM – Acellular Dermal Matrix, STSG – Split thickness skin graft.

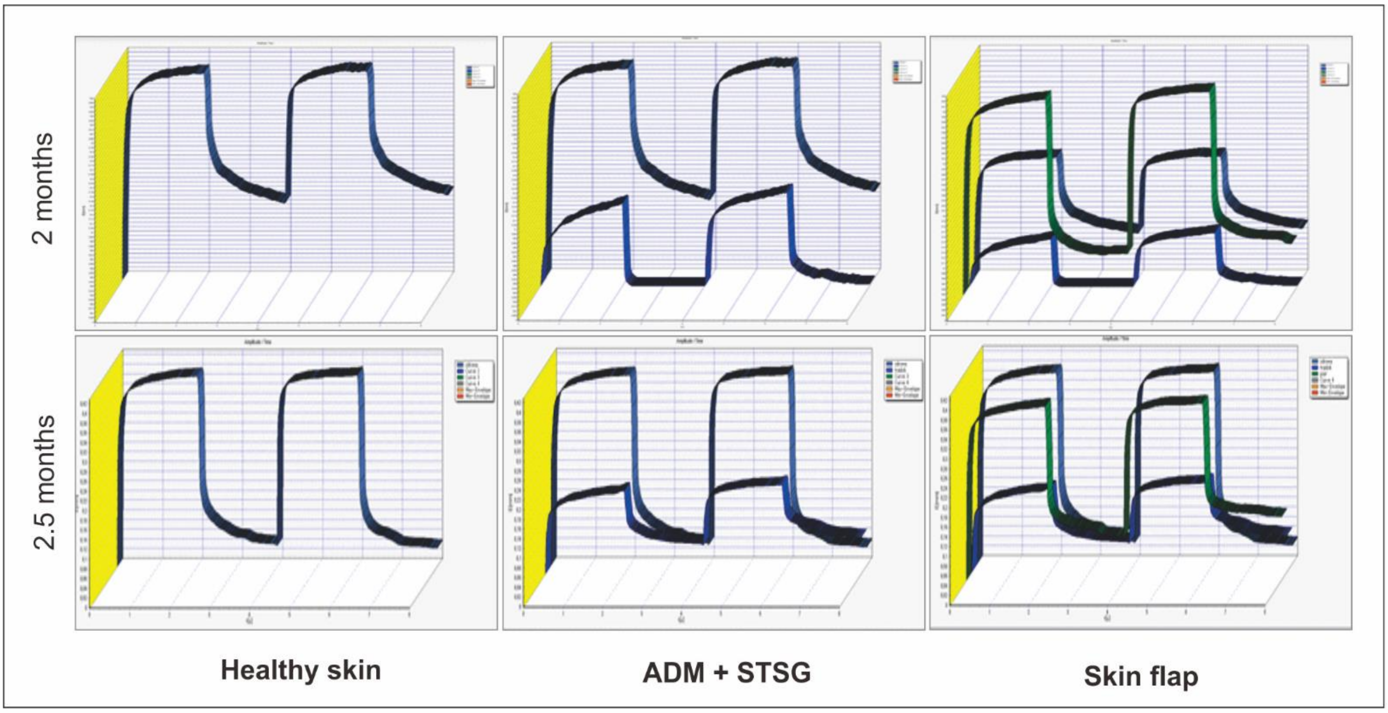

Figure S6. Cutometer measurements of healthy skin, ADM + STSG and skin flap of case 1. Legend : ADM – Acellular Dermal Matrix, STSG – Split thickness skin graft.

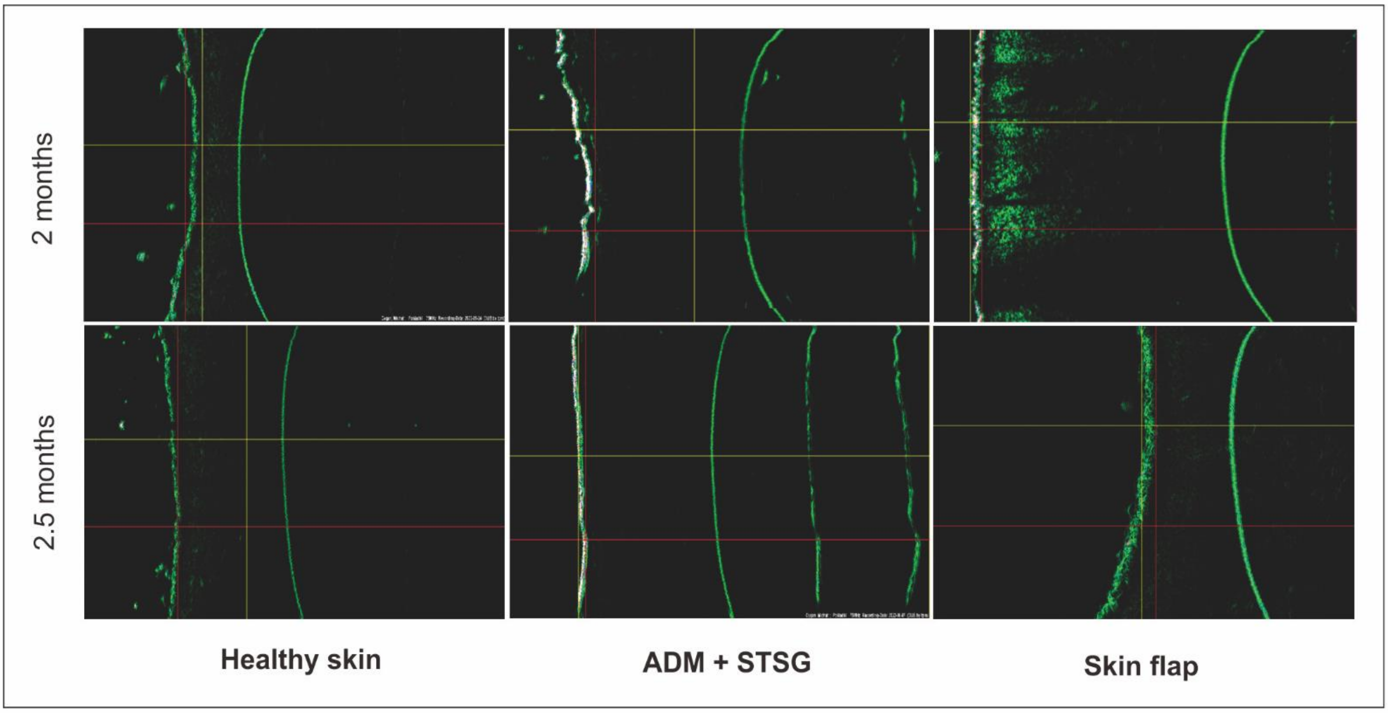

Figure S7. Skin ultrasound images (DUB SkinScanner v5.31 Dubai Silicon Oasis, Dubai UAE) of case 1 – skin flap, ADM and STSG and healthy skin in 2 months and 2.5 months after surgery. Legend: ADM – Acellular Dermal Matrix, STSG – Split thickness skin graft.

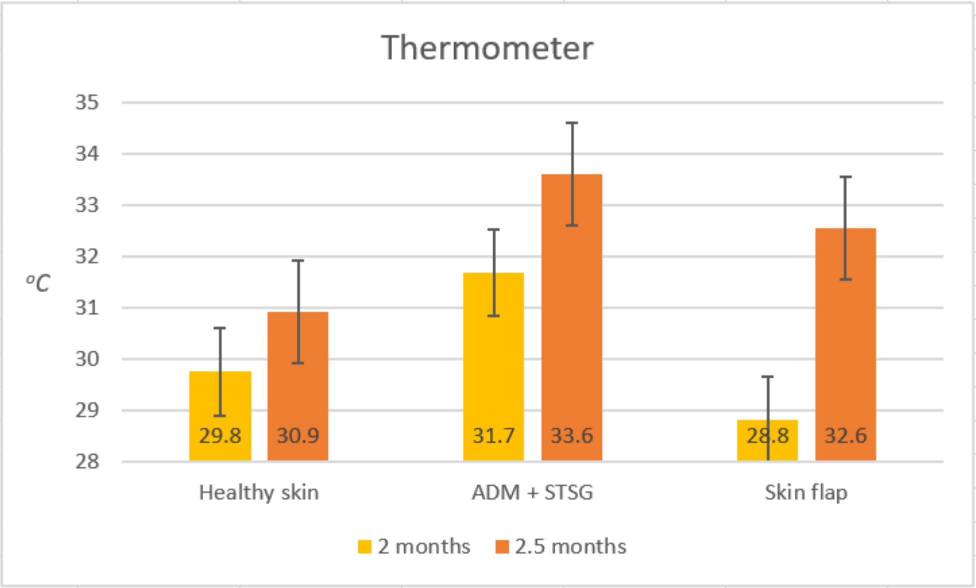

Figure S8. Temperature measurements of healthy skin, ADM + STSG and skin flap of case 2. Legend : ADM – Acellular Dermal Matrix, STSG – Split thickness skin graft.

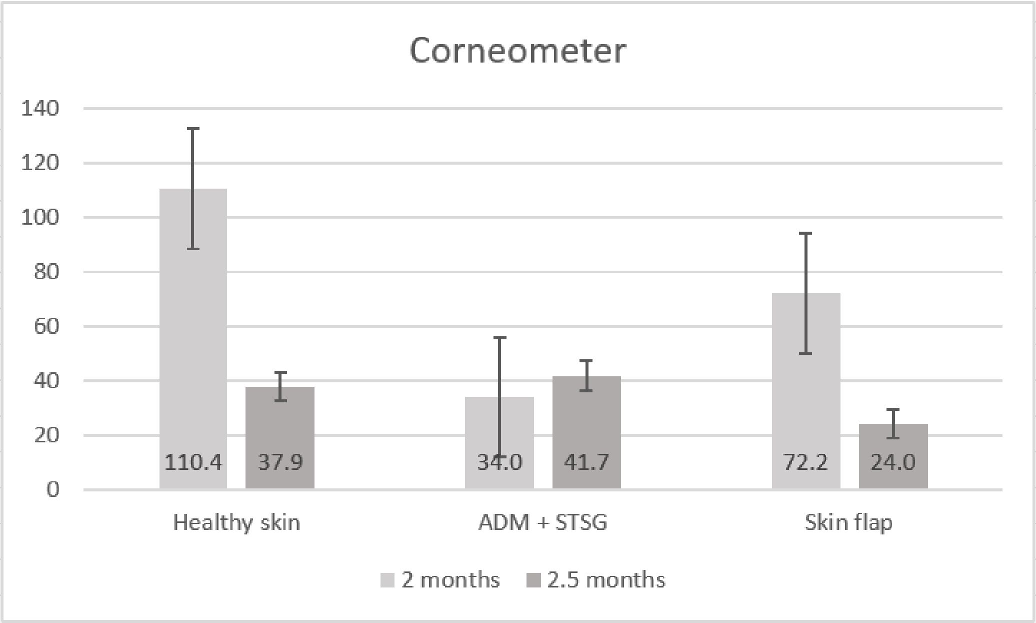

Figure S9. Corneometer measurements of healthy skin, ADM + STSG and skin flap of case 2. Legend : ADM – Acellular Dermal Matrix, STSG – Split thickness skin graft.

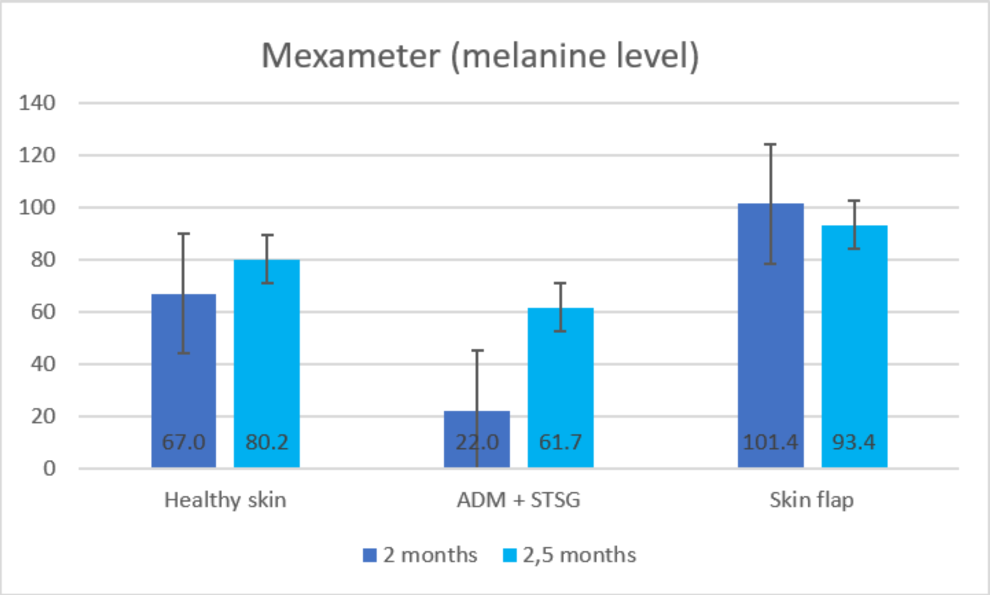

Figure S10. Mexameter (melanine level) measurements of healthy skin, ADM + STSG and skin flap of case 2. Legend : ADM – Acellular Dermal Matrix, STSG – Split thickness skin graft.

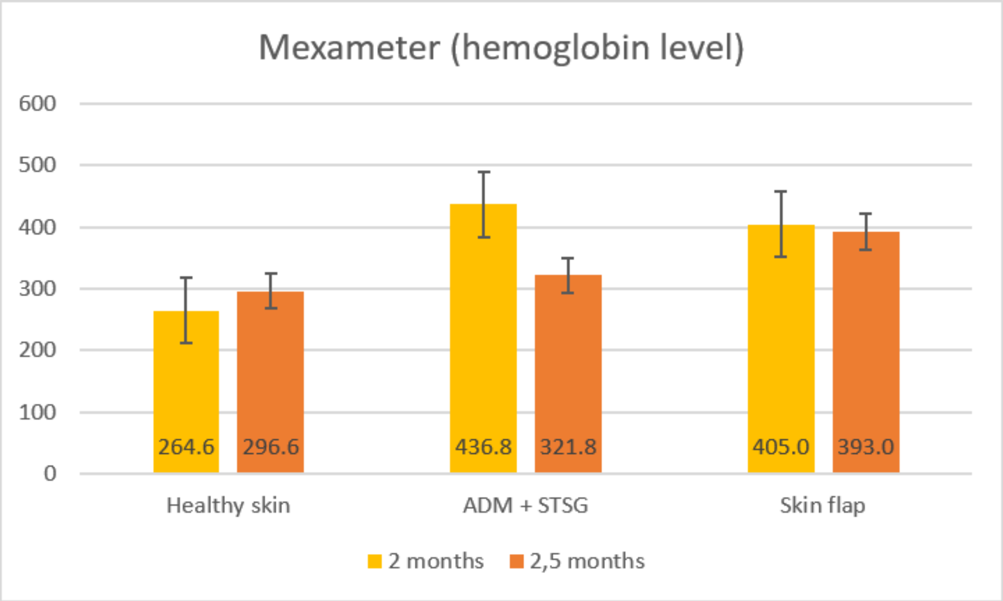

Figure S11. Mexameter (hemoglobine level) measurements of healthy skin, ADM + STSG and skin flap of case 2. Legend : ADM – Acellular Dermal Matrix, STSG – Split thickness skin graft.

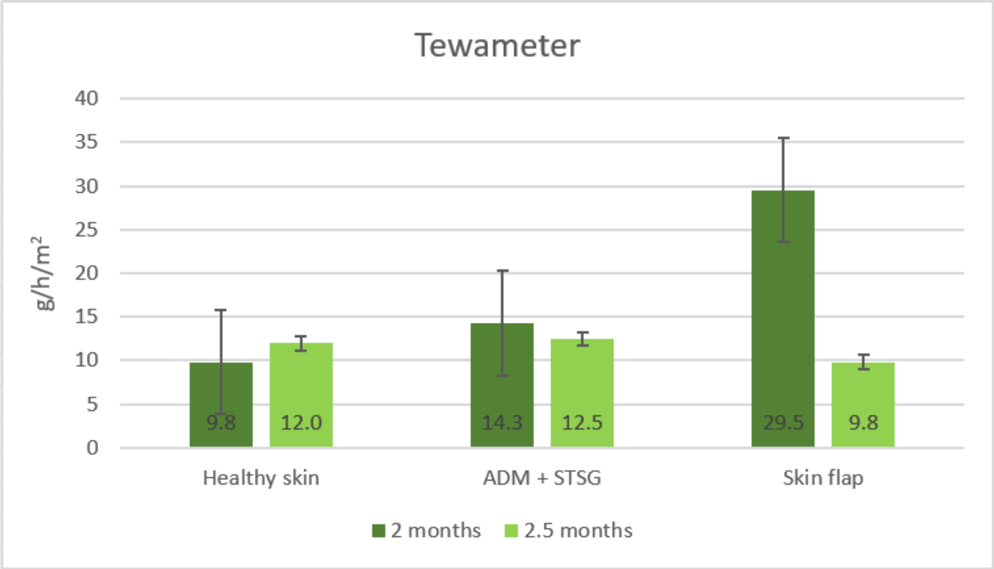

Figure S12. Tewameter measurements of healthy skin, ADM + STSG and skin flap of case 2. Legend : ADM – Acellular Dermal Matrix, STSG – Split thickness skin graft.

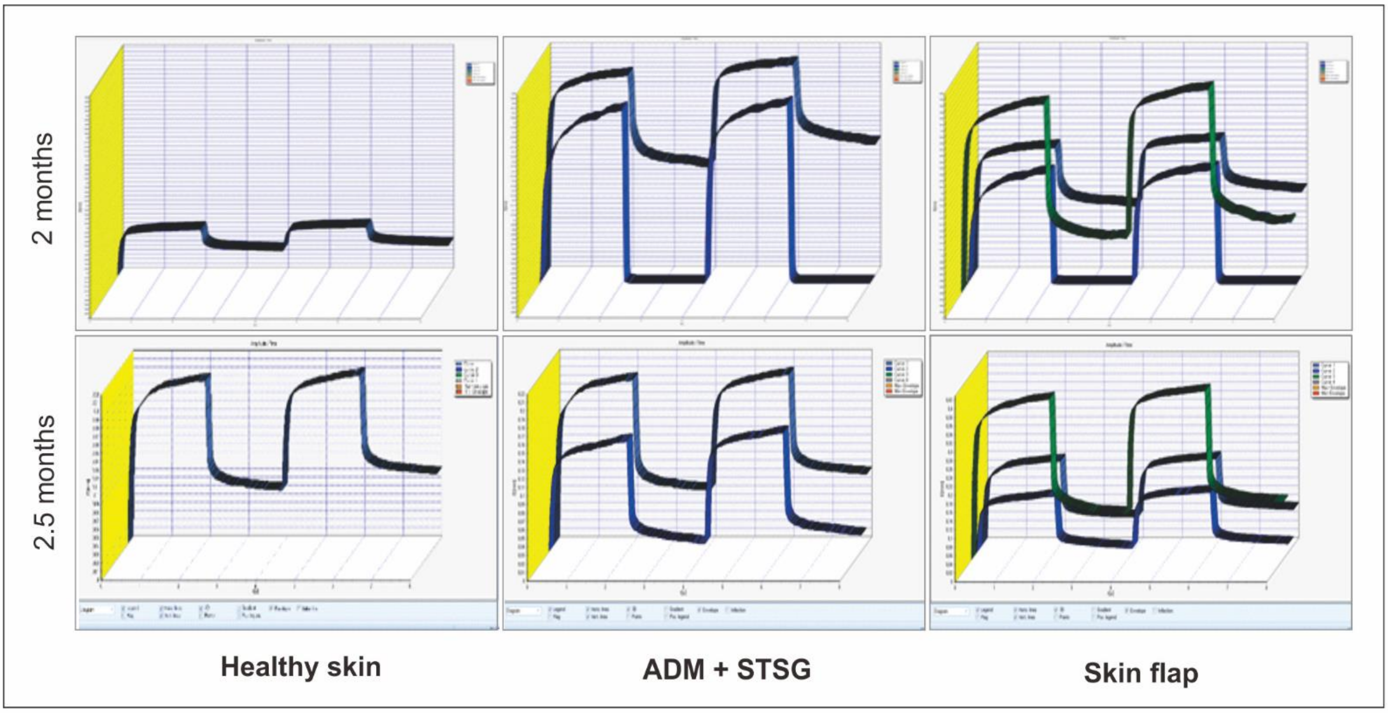

Figure S13. Cutometer measurements of healthy skin, ADM + STSG and skin flap of case 2. Legend : ADM – Acellular Dermal Matrix, STSG – Split thickness skin graft.

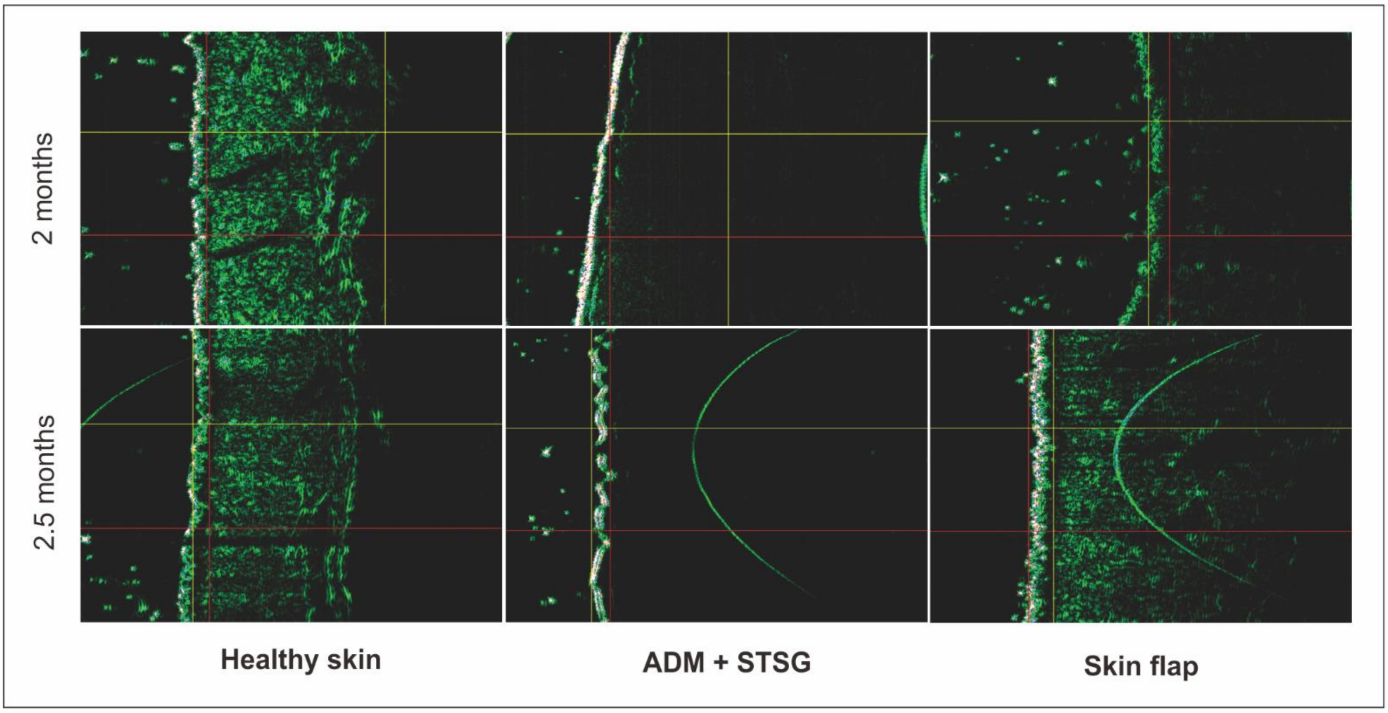

Figure S14. Skin ultrasound images (DUB SkinScanner v5.31 Dubai Silicon Oasis, Dubai UAE) of case 2 – skin flap, ADM and STSG and healthy skin in 2 months and 2.5 months after surgery. Legend : ADM – Acellular Dermal Matrix, STSG – Split thickness skin graft.

Table S1. Skin ultrasound results (DUB SkinScanner v5.31 Dubai Silicon Oasis, Dubai UAE) of case 1 – skin flap, ADM and STSG and healthy skin in 2 months and 2.5 months after surgery.

**Parameter**	**2 months**			**2.5 months**		
	healthy skin	ADM + STSG	skin flap	healthy skin	ADM + STSG	skin flap
Thickness of the epidermis (µm)	180	135	135	216	141	178
Epidermal density (%)	7.40	23.07	28.20	5.78	28.30	7.37
Thickness of the dermis (µm)	1344	1162	1500	1109	1032	1032
Density of the dermis (%)	2.69	0.92	3.51	2.69	0.97	0.97

Legend : ADM – Acellular Dermal Matrix, STSG – Split thickness skin graft.

Table S2. Skin ultrasound results (DUB SkinScanner v5.31 Dubai Silicon Oasis, Dubai UAE) of case 2 – skin flap, ADM and STSG and healthy skin in 2 months and 2.5 months after surgery.

**Parameter**	**2 months**			**2.5 months**		
	healthy skin	ADM + STSG	skin flap	healthy skin	ADM + STSG	skin flap
Thickness of the epidermis (µm)	159	150	188	137	198	176
Epidermal density (%)	22.35	26.37	6.10	12.92	21.02	25.26
Thickness of the dermis (µm)	1529	1206	1273	1273	844	1219
Density of the dermis (%)	6.36	1.51	1.12	5.57	1.53	4.68

Legend: ADM – Acellular Dermal Matrix, STSG – Split thickness skin graft.
